# Rejuvenation of corticospinal neurons enhances rehabilitation-associated corticospinal tract axon sprouting and functional recovery post photothrombotic ischemic stroke in mice

**DOI:** 10.1016/j.gendis.2025.102000

**Published:** 2025-12-22

**Authors:** Bin Sun, Li Sun, Lixiang Zhang, Xinyu Xue, Qiuyan Tian, Lei Wu, Mei Li, Jian Huang, Hong Ni, Lixiao Xu, Chenxi Feng, Jing Ren, Hongliang Huo, Xia Zhang, Xing Feng, Wenhao Zhou, Wanliang Guo, Yaobo Liu, Rong Ju, Zhenlang Lin, Xiaofeng Yang, Xin Ding

**Affiliations:** a Soochow Key Laboratory of Prevention and Treatment of Child Brain Injury, Children's Hospital of Soochow University, Suzhou, Jiangsu 215025, China; b Department of Maternal and Child Health, Suzhou Industrial Park Center for Disease Control and Prevention, Suzhou, Jiangsu 215100, China; c School of Basic Medical Sciences, Medical College of Soochow University, Suzhou, Jiangsu 215025, China; d Department of Rehabilitation, Children's Hospital of Soochow University, Suzhou, Jiangsu 215025, China; e Department of Neurology and Clinical Research Center of Neurological Disease, The Second Affiliated Hospital of Soochow University, Suzhou, Jiangsu 215025, China; f Guangzhou Women and Children's Medical Center, Guangzhou Medical University, Guangzhou, Guangdong 511436, China; g Radiology Department, Children's Hospital of Soochow University, Suzhou, Jiangsu 215025, China; h Jiangsu Key Laboratory of Drug Discovery and Translational Research for Brain Diseases, Institute of Neuroscience, Soochow University, Department of Rehabilitation Medicine and Neurology, The Fourth Affiliated Hospital of Soochow University, Suzhou, Jiangsu 215123, China; i Chengdu Women's and Children's Central Hospital, School of Medicine, University of Electronic Science and Technology of China, Chengdu, Sichuan 610073, China; j Department of Neonatology, The Second Hospital and Yuying Children's Hospital of Wenzhou Medical University, Wenzhou, Zhejiang 325027, China

**Keywords:** Corticospinal neurons, CST axon sprouting, Functional recovery, Klf4, Oct4, Photothrombotic stroke, Rehabilitation, Sox2

## Abstract

Spontaneous recovery following an ischemic stroke is often limited, largely attributed to age-related decline in neuroplasticity. To overcome this, we demonstrated that ectopic expression of a cocktail of transcriptional factors (Oct4, Sox2, and Klf4, referred to as OSKTFs) reset developmental decline of epigenetic signatures in adult corticospinal neurons, without affecting their spinal projection patterns and function in controlling skilled locomotion. Corticospinal expression of OSKTFs had moderate effects on promoting collateral sprouting of the corticospinal tract axons and recovery of skilled motor function following a photothrombotic stroke. When combined with task-dependent rehabilitative training, OSKTFs treatment significantly enhanced its efficacy, suggesting that rejuvenating corticospinal neurons substantially amplifies the beneficial outcomes of rehabilitative training. Mechanistically, pharmacological perturbations and intersectional chemogenetic inhibition establish that both axon sprouting and functional recovery require mTOR activation and are mediated by newly sprouted corticospinal tract axons. Together, these findings identify a novel strategy to rejuvenate adult corticospinal neurons, which improves the otherwise modest benefits typically gained from rehabilitative training after traumatic brain injuries.

## Introduction

Due to limited recovery after acute injury, the majority of patients with ischemic stroke are left with various degrees of neurological deficits.^1^^,^^2^ In most clinical cases, limited recovery occurs during the first 3 months after stroke.^3^^,^^4^ Notably, the spontaneous recovery is driven by residual neural plasticity that leads to axon sprouting, synaptogenesis, and circuit remapping that enables functional compensation.^5^ However, the neuroplasticity of the central nervous system (CNS) exhibits considerable age-related decline post stroke.^6^, ^7^, ^8^, ^9^

Rehabilitative training is widely adopted in the clinic to achieve functional recovery.^1^^,^^5^^,^^10^ The rationale of rehabilitative training is based on the Hebb theory, which predicts simultaneous pre- and post-synaptic activities that will facilitate synaptogenesis and ultimately lead to the formation of new circuits.^11^ However, the effectiveness of rehabilitative training is highly dependent on the level of neuroplasticity and, consequently, is limited in aged patients. Thus, a logical strategy to improve outcomes of rehabilitative training is to identify avenues that are capable of rejuvenating adult neurons in the CNS.

Epigenetic changes are well recognized as hallmarks of ageing.^12^, ^13^, ^14^ The transcription factor-based cellular reprogramming can refresh the epigenetic landscape and thus presents an innovative method for the rejuvenation of aging cells.^15^ For instance, *ex vivo* expression of four transcriptional factors, octamer-binding transcription factor 4 (Oct4), sex-determining region Y-box 2 (Sox2), Kruppel-like factor 4 (Klf4), and c-Myc, can completely reset the epigenetic marks acquired during development, which enables the transformation of specialized somatic cells back into a state of pluripotency.^16^ In light of this, recent studies have shown that overexpression of Oct4, Sox2, and Klf4 (referred to as OSKTFs) reverses epigenetic changes in aged retinal ganglion cells and enables them to regrow their injured axons, a process typically absent in the mature mammalian CNS.^17^^,^^18^ However, whether the same strategy rejuvenates corticospinal neurons, the neural substrate for top-down motor control,^19^^,^^20^ remains unclear.

In the current study, we first showed that unilateral photothrombotic stroke ablated corticospinal neurons, leading to severe impairments in skilled but not gross motor function. We further demonstrated that expression of OSKTFs in corticospinal neurons partially rescued the developmental decline of major epigenetic regulators. Ectopic expression of OSKTFs in corticospinal neurons had minimal impact on corticospinal tract (CST) axons' spinal termination and function in intact animals but moderately promoted the collateral outgrowth of the CST axons in the cervical spinal cord and skilled motor recovery in animals with photothrombotic stroke. OSKTFs expression synergized with rehabilitative training through enhanced mTOR activity, producing additive benefits on CST collateral sprouting and skilled locomotion recovery. Mechanistically, the observed axon sprouting and functional recovery depend on mTOR activation and are driven by newly formed CST collaterals. Taken together, our study revealed an effective avenue to rejuvenate corticospinal neurons, thereby providing new thoughts to optimize the otherwise modest effects of rehabilitative training that is widely used for treating patients with traumatic CNS injuries.

## Materials and methods

### Animals

All surgical procedures and behavioral measurements were approved by the Animal Ethical Committee of Soochow University (No. XD-2020-1). C57BL/6 mice of both sexes were used.

**Figure 1:**
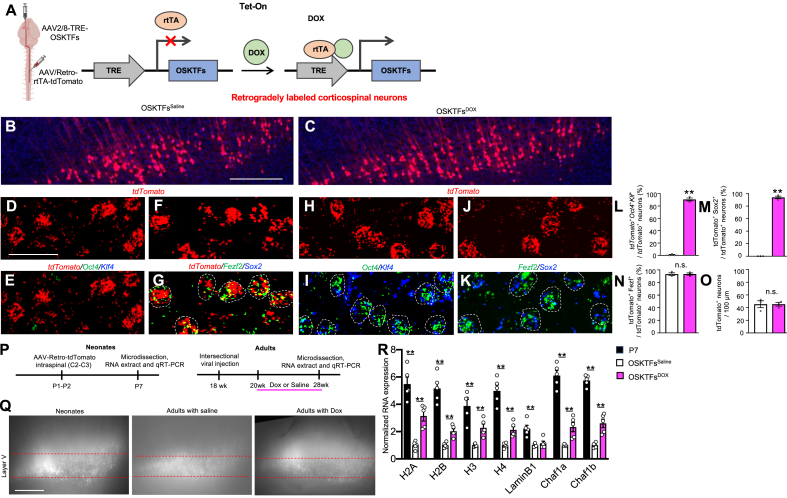
Ectopic expression of OSKTFs in retrogradely labeled corticospinal neurons. **(A)** Schematic diagrams of the experimental design and the Tet-on system. **(B, C)** Representative images of transverse brain sections showing retrogradely labeled corticospinal neurons in mice treated with saline or doxycycline (Dox). Scale bar: 100 μm. **(D**–**K)** Representative high-magnitude images of layer V sensorimotor cortex showing fluorescent *in situ* hybridization of *tdTomato* and transcriptional factors *Oct4*, *Sox2*, *Klf4*, and *Fezf2* in mice treated with saline or Dox. Scale bar: 50 μm. **(L–O)** Quantification of colocalization between *tdTomato* and *Oct4/Klf* (L), *Sox2* (M), *Fezf2* (N), and *tdTomato* neuronal densities (O) in animals treated with saline (*n* = 3) or Dox (*n* = 3). ∗∗*P* < 0.01; n.s., no statistical significance; Student's *t*-test. **(P, Q)** Neonates (P1) or adult (18 weeks) animals received intraspinal AAVRetro-tdTomato or intersectional injection for OSKTFs expression in corticospinal neurons. Mice were euthanized at P7 or 28 weeks with an 8-week Dox or saline treatment (P). Layer V of the sensorimotor cortex was microdissected under an epi-fluorescent microscope (Q) for measuring RNA contents. Scale bar: 500 μm. **(R)** Relative RNA expression of different histone proteins and epigenetic modulators in P7 mice (*n* = 5 biological replicates) and adult mice treated with saline (*n* = 5 biological replicates) or Dox (*n* = 5 biological replicates). RNA expression levels were first normalized to those of GAPDH and then to those of adult animals treated with saline. ∗∗*P* < 0.01; one-way ANOVA followed by the Bonferroni correction.

**Figure 2:**
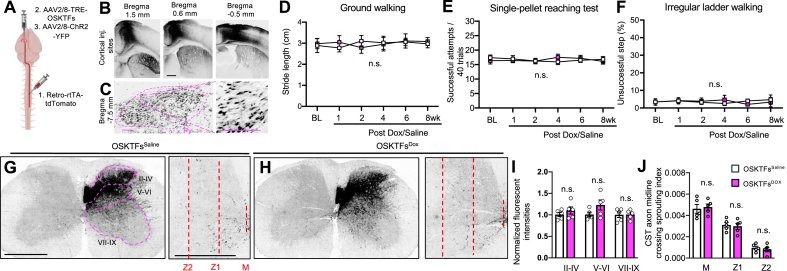
Effects of OSKTFs expression in corticospinal neurons on neuronal integrity and skilled motor function. **(A)** Schematic of the experimental design. **(B, C)** Representative transverse brain sections (B) showing intersectionally labeled corticospinal neurons and the pyramidal (Py) with a high-magnification image illustrating individual axons derived from corticospinal neurons (C). Scale bars in B: 500 μm; in C: 100 μm. **(D–F)** Performance of gross locomotion, single pellet reaching, and irregular ladder walking in mice treated with saline (*n* = 9) or doxycycline (Dox) (*n* = 10) at different time points post treatment. n.s., no statistical significance; repeated measures of two-way ANOVA. **(G, H)** Representative transverse images with high-magnitude ones of the cervical spinal cord showing CST axons in mice treated with saline or Dox. Scale bar: 500 μm. The dotted line in Figure G illustrates the contour of distinct laminae. **(I, J)** Quantification of the CST axon innervation in distinct laminae on the contra-lateral side (I) and its cross-midline sprouting (J) in mice treated with saline (*n* = 5) or Dox (*n* = 5). n.s., no statistical significance; Student's *t*-test.

**Figure 3:**
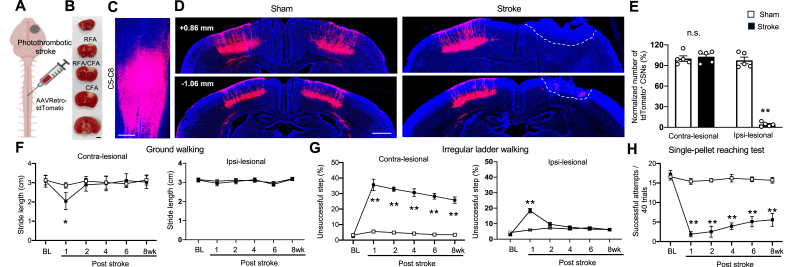
Effects of photothrombotic stroke on corticospinal neurons and locomotion behaviors. **(A)** Schematic drawing of the experimental design (left) and a representative horizontal image (right) of the spinal cord showing the injection site (blue: DAPI; red: AAV-retro-tdTomato). **(B)** TTC staining of representative transverse brain sections from a mouse with a photothrombotic stroke of forelimb areas. RFA: rostral forelimb area; CFA: caudal forelimb area. **(C–E)** Representative spinal cord (C) and transverse brain images (D) stained for tdTomato showing the infection in the cervical spinal cord (C) and retrogradely labeled corticospinal neurons (D) in mice with sham injury (*n* = 8) or photothrombotic stroke (*n* = 8) with quantifications (E). n.s., no statistical significance; ∗∗*P* < 0.01; Student's *t*-test. **(F–H)** Performance of gross locomotion, single pellet reaching, and irregular ladder walking in mice with sham injury (*n* = 8) or photothrombotic stroke (*n* = 8) at different time points after stroke. ∗∗*P* < 0.01 and ∗*P* < 0.05; repeated measures of two-way ANOVA followed by the Bonferroni correction. Scale bars: 1 mm.

**Figure 4:**
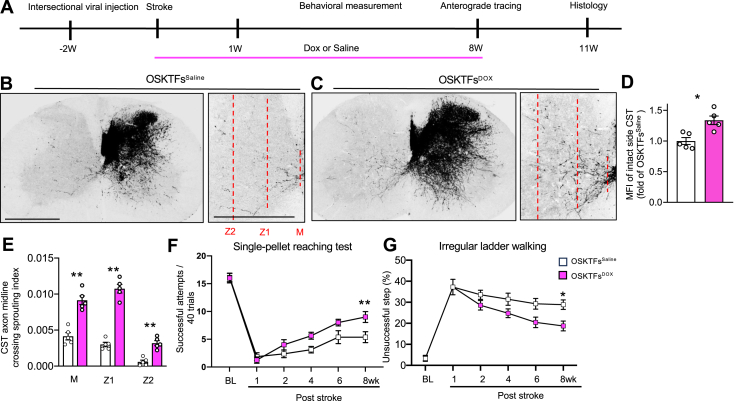
Effects of corticospinal expression of OSKTFs on the CST axon sprouting and locomotion recovery after photothrombotic stroke. **(A)** The timeline of the experimental design. **(B, C)** Representative transverse images with high-magnitude ones of the cervical spinal cord showing CST axons in stroke mice treated with saline or doxycycline (Dox). Scale bar: 500 μm. **(D, E)** Quantification of normalized fluorescent intensities on the intact side (D) and the CST axon sprouting indexes at different distances to the midline on the denervated side (E) in stroke mice treated with saline (*n* = 5) or Dox (*n* = 5). ∗∗*P* < 0.01 and ∗*P* < 0.05; Student's *t*-test. **(F, G)** Performance of single pellet retrieval and irregular ladder walking tasks in stroke mice treated with saline (*n* = 9) or Dox (*n* = 9) at different time points after stroke. ∗∗*P* < 0.01 and ∗*P* < 0.05; repeated measures of ANOVA followed by the Bonferroni correction.

**Figure 5:**
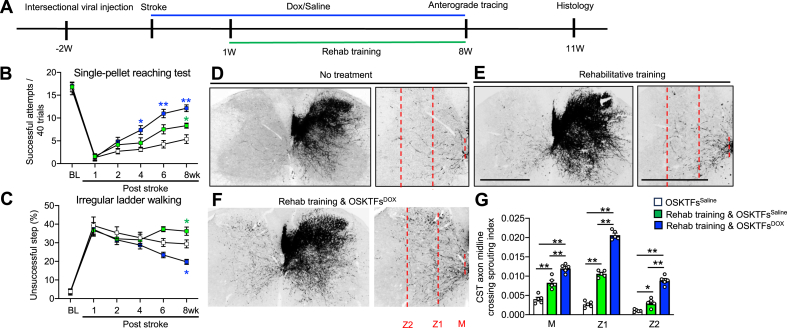
Effects of corticospinal expression of OSKTFs in combination with rehabilitative training on the CST axon sprouting and locomotion recovery after photothrombotic stroke. **(A)** The timeline of the experimental design. **(B, C)** Performance of single pellet retrieval and irregular ladder walking tasks at different time points after stroke in mice with no treatment (*n* = 12), rehabilitative training (*n* = 12), and rehabilitative training with OSKTFs^Dox^ treatment (*n* = 12), respectively. Blue ∗∗*P* < 0.01 and ∗*P* < 0.05 (Rehab + OSKTFs versus no treatment); green ∗*P* < 0.05 (Rehab training versus no treatment); repeated measures of two-way ANOVA followed by the Bonferroni correction. **(D**–**F)** Representative transverse images with high-magnitude ones of the cervical spinal cord showing CST axons in stroke mice with no treatment, rehabilitative training, or rehabilitative training with OSKTFs^Dox^ treatment. Scale bar: 500 μm. **(G)** Quantification of the CST axon sprouting indexes at different distances to the midline in stroke mice with no treatment (*n* = 5), rehabilitative training (*n* = 5), or rehabilitative training with OSKTFs^Dox^ treatment (*n* = 5). ∗∗*P* < 0.01 and ∗*P* < 0.05; one-way ANOVA followed by the Bonferroni correction.

**Figure 6:**
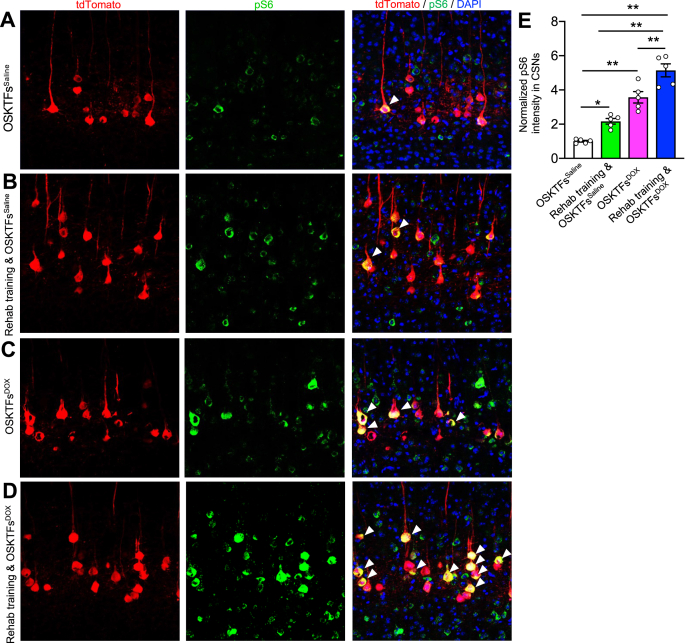
Effects of corticospinal expression of OSKTFs in combination with rehabilitative training on mTOR activity in corticospinal neurons. **(A–D)** Representative images showing immunostaining of retrogradely labeled corticospinal neurons (red) and pS6 (green) in mice with different treatments. **(E)** Quantification of the relative fluorescence intensity of pS6 in retrogradely labeled corticospinal neurons in mice with different treatments. Relative fluorescent intensities were normalized to those in animals with no treatment. ∗∗*P* < 0.01 and ∗*P* < 0.05; one-way ANOVA followed by the Bonferroni correction.

**Figure 7:**
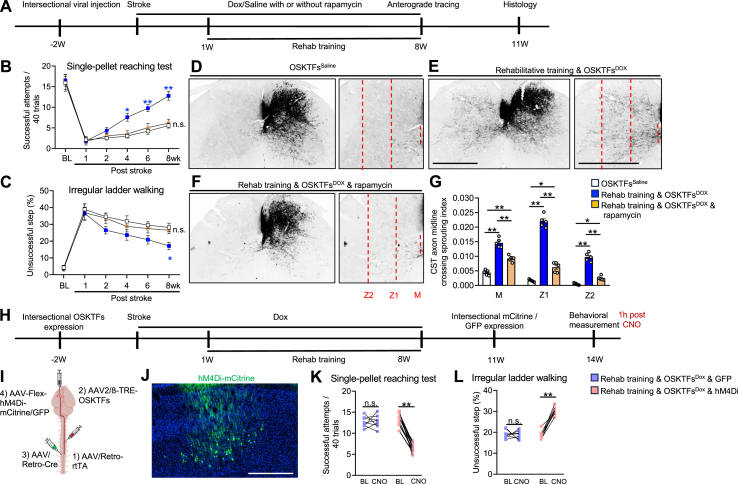
Effects of rapamycin and chemogenetic silencing on CST sprouting and functional recovery with combined treatment. **(A)** The timeline of the experimental design of mTOR inactivation with OSKTFs treatment and rehabilitation. **(B, C)** Performance of single pellet retrieval (B) and irregular ladder walking (C) tasks at different time points after stroke in OSKTFs mice treated with saline (*n* = 12), with doxycycline (Dox) and rehabilitative training (*n* = 12), or with Dox/rapamycin and rehabilitative training (*n* = 12). Blue ∗∗*P* < 0.01 and ∗*P* < 0.05 (Rehab + OSKTFs versus saline); n.s., no statistical significance (Rehab + OSKTFs & rapamycin *vs* saline); repeated measures of two-way ANOVA followed by the Bonferroni correction. **(D**–**G)** Representative transverse images with high-magnitude ones of the cervical spinal cord showing CST axons (D–F), along with quantification of the CST axon sprouting indexes (G) at different distances to the midline in OSKTFs stroke mice treated with saline (*n* = 5), rehabilitative training with Dox (*n* = 5), or rehabilitative training with Dox and rapamycin (*n* = 5). ∗∗*P* < 0.01 and ∗*P* < 0.05; one-way ANOVA followed by the Bonferroni correction. Scale bar: 500 μm. **(H)** The timeline of the experimental design of chemogenetic inactivation of sprouted corticospinal neurons with OSKTFs treatment and rehabilitation. **(I, J)** Schematic drawing of experimental design (I) and a representative image (J) showing hM4Di-mCitrine or GFP expression in corticospinal neurons with midline-crossing collateral sprouting. Scale bar: 500 μm. **(K, L)** Performance of single pellet retrieval (K) and irregular ladder walking (L) tasks in OSKTFs^Dox^ animals with intersectional GFP (*n* = 8) or hM4Di-mCitrine (*n* = 8) expression before and 1 h after CNO. n.s., no statistical significance; ∗∗*P* < 0.01; paired *t*-test.

For experiments shown in Figure 1, Figure 2, intersectional viral injection (AAVRetro-rtTAV16-tdTomato into the spinal cord and AAV-TRE-OSKTFs into the sensorimotor cortex) was performed at 18 weeks of age. To induce sustained OSKTFs expression, doxycycline or saline was administered for 8 weeks, followed by histological and behavioral assessments. In addition, to examine neonatal expression of histone proteins and epigenetic modulators, neonatal mice received cervical spinal injections of AAVRetro-tdTomato at postnatal day 1 (P1) as indicated in Figure 1P. For experiments shown in Figure 3, Figure 4, Figure 5, Figure 6, Figure 7, the same intersectional injection protocol was performed at 18 weeks of age. Photothrombotic stroke was induced at 20 weeks, and mice then received 8 weeks of doxycycline or saline treatment.

### Targeting corticospinal neurons

We used different protocols to label corticospinal neurons of neonatal or adult animals. For adult corticospinal neurons, C57 mice were anesthetized (2% isoflurane with oxygen at a flow rate of 0.5 L/min) and placed in a stereotactic frame. After laminectomy, AAV2Retro-tdTomato (titer adjusted to 1 × 10^13^ copies/mL, generated by BrainVTA, Wuhan, China) was bilaterally injected into the cervical (C5–C8) spinal cord (0.3, 0.5 ML; 0.35 depth, 100 nL per injection site). To estimate the ablation of corticospinal neurons due to unilateral photothrombotic stroke, we prepared transverse sections of the brain (60 μm) and took images using a confocal laser scanning microscope (LSM700, Zeiss). The numbers of retrogradely traced corticospinal fibers of both sides were blindly quantified per section. Five sections crossing from the rostral to caudal cortex, spanning both the rostral (RFA) and the caudal (CFA) forelimb areas, were included for quantification per individual animal.

To target neonatal corticospinal neurons, P1 pups received hypothermic anesthesia and were placed under an ultrasound machine (Vevo 770, FUJIFILM VisualSonics)^21^ for guided spinal injection of AAVRetro-tdTomato (titer adjusted to 1 × 10^13^ copies/mL, generated by BrainVTA, Wuhan, China).

### Ectopic expression of OSKTFs in corticospinal neurons

To induce ectopic expression of OSKTFs in adult corticospinal neurons, we adopted a two-step intersectional labeling strategy. The first step involves retrograde labeling of corticospinal neurons innervating the cervical spinal cord. We used the same protocol as described above by replacing AAV2Retro-tdTomato with an AAV2Retro-rtTAV16-2A-tdTomato (titer adjusted to 1 × 10^13^ copies/mL, generated by BrainVTA, Wuhan, China). Two weeks later, we performed subsequent stereotaxic cortical injections (AAV-TRE-Oct4-Sox2-Klf4, titer adjusted to 1 × 10^13^ copies/mL, generated by BrainVTA, Wuhan, China) to intersectionally express Tet-On OSKTFs in retrogradely targeted corticospinal neurons. The vector contains a tight tetracycline response element (TRE) positioned upstream of the polycistronic mouse OSKTFs coding sequences. To further enhance OSKTFs' expression efficiency, the commonly used WPRE and human growth hormone poly(A) signal were replaced with an SV40 poly(A) signal (vector sequence and map were provided in Figure S1). The injection coordinates were: +1.5, +1, +0.5, 0, and −0.5 mm anterior to the bregma; 1.6 and 2.2 mm lateral to the midline, with a depth of 0.6 mm; 100 nL per injection site.

To induce OSKTFs expression, doxycycline (2 mg/mL) was administered in the drinking water continuously for 8 weeks. The drinking water containing doxycycline was refreshed whenever the bottles were consumed or at least every 3 days to ensure consistent dosing. To inhibit mTOR activity, rapamycin (Sigma, 553210) was diluted from a stock solution (20 mg/mL in ethanol) to 1 mg/mL in 5% Tween-80 and 5% polyethylene glycol 400 in phosphate-buffered saline (PBS; vehicle), and administered by intraperitoneal injection at 6 mg/kg every other day together with doxycycline treatment for 8 weeks.

### Assessment of OSKTFs induction and effect on epigenetic modulators in retrogradely labeled corticospinal neurons

To examine ectopic induction of Oct4, Sox2, and Klf4 in adult retrogradely labeled (tdTomato^+^) corticospinal neurons, we i) prepared transverse brain sections (15 μm) crossing the RFA and CFA regions in animals with OSKTFs^Dox^ or OSKTFs^saline^ treatment; and ii) performed *in situ* hybridization using the RNAscope® Multiplex Fluorescent Detection Kit v2 (Advanced Cell Diagnostics) following the manufacturer's guidelines. Due to technical challenges in combining fluorescent immunohistochemistry with *in situ* hybridization, we identified retrogradely labeled corticospinal neurons by detecting tdTomato RNAs, an approach that enables robust detection of virally labeled neurons.^22^^,^^23^ RNAscope probes used were: i) *tdTomato* (Cat No. 317041); ii) *Mm-Pou5f1(Oct4)-C2* (Cat No. 426961-C2); iii) *Mm-Sox2-C2* (Cat No. 401041-C2); iv) *Mm-Klf4-C3* (Cat No. 426711-C3); v) *Mm-Fezf2-C3* (Cat No. 313301-C3). Notably, because the kit allows a maximum of 3 channels for co-staining, we performed *in situ* hybridization of *tdTomato/Oct4/Klf4* and *tdTomato/Sox2/Fezf2* (FEZ family zinc finger 2) in adjacent sections. To quantify co-expression of *Oct4/Klf4*, *Sox2*, and *Fezf2* in retrogradely labeled corticospinal neurons (*tdTomato*^*+*^), we chose five sections that spanned the RFA and CFA regions (1.5 mm to −0.5 mm to the bregma, 500 μm/section) for individual animals. Co-localization of different probes with tdTomato among all tdTomato corticospinal neurons (∼70–130 per section) was then blindly quantified for individual sections. Colocalization ratios calculated from 5 sections were then averaged for each animal and used as one data point.

To examine the effects of OSKTFs on the expression of epigenetic modulators, mice with retrograde labeling of corticospinal neurons or ectopic expression of OSKTFs/tdTomato in corticospinal neurons were euthanized at P7 and 28 weeks, respectively. Brains were quickly removed and sliced using a 1 mm coronal matrix. Next, under a fluorescent dissection microscope (Zeiss), the region that contains the layer V tdTomato^+^ corticospinal neurons from sensorimotor cortices was quickly dissected out using a needle blade microknife. Pooled tissues from 3 mice (5 biological replicates for each condition) were homogenized, and total RNAs were extracted with the TRIzol Reagent (Thermo Fisher Scientific), reverse-transcribed (Superscript III, Invitrogen), and quantified with the Sybr green-based quantitative PCR (CFX Connect, BioRad).^24^ Gapdh RNA levels were used for internal normalization between samples. Next, the relative expression of distinct RNAs was further normalized to that in an adult with ectopic tdTomato expression in corticospinal neurons. qRT primers used were: Histone H2A Forward: GCGACAACAAGAAGACGCGCAT, Reverse: CTGGATGTTGGGCAGGACGCC; Histone H2B Forward: AAGAAGGACGGCAAGAAGCGCA, Reverse: CGCTCGAAGATGTCGTTCACGA; Histone H3.1/3.2 Forward: GAAGAAGCCTCACCGCTACCG, Reverse: GGTTGGTGTCCTCAAACAGACCC; Histone H4 Forward: AACATCCAGGGCATCACCAAGC, Reverse: GTTCTCCAGGAACACCTTCAGC; LaminB1 Forward: CCGGCCTCAAGGCTCTCTA, Reverse: TGCCGCCTCATACTCTCGAA; Chaf1a Forward: CGCGGACAGCCGCGGCCGTGGATTGC, Reverse: GTGTCTTCCTCAACTTTCTCCTTGG; Chaf1b Forward: CACCGCCGTCAGGATCTGGAAGTTGG, Reverse: GGCTCCTTGCTGTCATTCATCTTCCAC; GAPDH Forward: CATCACTGCCACCCAGAAGACTG, Reverse: ATGCCAGTGAGCTTCCCGTTCAG.

### Establishment of a cortical photothrombotic ischemic stroke model

To establish a cortical photothrombotic ischemic stroke model, we adapted previously reported protocols.^20^^,^^25^ In brief, we performed the intraperitoneal injection of Rose Bengal (10 mg/kg in a 5 mg/mL saline solution) 10 min before light exposure. C57 mice were then anesthetized (2% isoflurane with oxygen at a flow rate of 0.5 L/min) and head fixed in a stereotactic frame. A small incision was made to expose the skull, followed by the attachment of an opaque circular template (diameter: 2 mm; center: AP: −0.5 mm, ML: 2.0 mm to the bregma)^19^^,^^25^ that covers both the rostral and caudal forelimb areas for targeted illumination. A cold light source (Zeiss, CL 1500HAL, 3000 K) was used to induce photothrombotic stroke in the defined area for 15 min. Sham-operated mice received the same procedures without exposure to the light source. Transverse brain sections (1 mm thickness) were stained with 4% 2,3,5-triphenyltetrazolium chloride (TTC) (Sigma) 24 h post-thrombotic stroke to confirm the infarct regions.

### Behavioral measurements

#### *Gross locomotion*

To assess the effects of photothrombotic stroke on gross locomotion, we recorded mice's walking in a narrow rectangular path (1 m length, ∼30 steps) using a video camera (GoPro). Mice were trained to walk from one end to the other end of a rectangular Plexiglas box as pre-test habituation. During the test day, the camera was mounted on a frame to obtain a lateral view of the walking through the narrow path. The tip of the forelimb toes was marked in white. With pre-labeled scales on the path, each forelimb stride length was manually measured by blindly watching the video. Mean forelimb stride of contra-cortical injection (Fig. 2) or both sides (Fig. 3) was measured at 1, 2, 4, 6, and 8 weeks after stroke.

#### *Skilled motor function*

We used two behavioral measurements to assess skilled motor function. Both measurements were dependent on corticospinal control of skilled locomotion.^19^^,^^20^^,^^26^

##### Irregular ladder walking

We first irregularly placed rungs (distances between rungs varied from 1 to 3 cm) onto a horizontal ladder (1 m length) and positioned the ladder on a stand 30 cm above the table surface. Before any tests, mice were trained to navigate the ladder for 15–20 trials. The trials were recorded using a GoPro camera and later blindly assessed for error rate/trial. A successful step is counted only if the mouse places the center of its forepaw on the rung with its digits closed. After training, an average error rate on the irregular ladder is between 10% and 15%. Mice with error rates larger than 20% were excluded from further manipulations. During the test day, mice were allowed to cross the ladder at 2 weeks before (baseline), or 1, 2, 4, 6, and 8 weeks post stroke and/or doxycycline administration. The error rates of either contra-lateral side to the injected cortex in intact animals (Fig. 2), both sides (Fig. 3), or the contra-lesional side after stroke (Figure 4, Figure 5) were then blindly quantified based on recorded videos.

##### *Single pellet retrieval task*

The single pellet retrieval task was performed with minor adaptations from the established protocols.^19^^,^^27^ In brief, overnight fasted mice were placed in a Plexiglas chamber (20 × 15 × 8.5 cm), which contains a slim vertical opening attached with a narrow platform on the front wall. For individual trials, we placed a single sugar pellet (dustless precision pellet, 20 mg, Bioserv). Mice were encouraged to grasp the pellet using their forepaws and put it into their mouths. Daily training contains up to 40 attempts within a 20-min window. The success rate was determined by dividing the number of successful retrievals by the total number of attempts. After a 7-to-10-day training session, most mice achieved a success rate between 0.3 and 0.4. Those with a success rate less than 0.25 were excluded from further functional manipulations. Throughout the training period, all mice were kept at no less than 90% of their normal free-feeding body weight. Notably, in most cases, mice exhibited forepaw laterality in the single pellet retrieval task. Therefore, the unilateral photothrombotic stroke was intentionally performed at the contralateral cortex of their preferred forepaws.

Daily training of the single pellet retrieval test was selected as the task-dependent rehabilitative training. According to previous protocols, we commenced the training at 10 days post stroke.^20^^,^^28^ Our established routine consisted of 40 trials each day, conducted seven days a week, continuing up to the eighth week following the injury.

All behavioral measurements were conducted before daily training, ensuring that the assessment of motor skills was not influenced by the training itself. Animals were tested at 2 weeks before (baseline), or 1, 2, 4, 6, and 8 weeks post stroke and/or doxycycline administration.

### Immunohistochemistry and quantification

Floating transverse brain sections crossing the RFA and CFA regions were prepared (60 μ m) using a cryostat (Leica), blocked (0.5% Triton-100, 1xPBS) at room temperature for 2 h, incubated with the 1st antibodies overnight, washed, and subsequently incubated with the 2nd antibodies for 2 h. The 1st antibody: rabbit-anti-pS6 [Cell signaling technology (4857), 1:200]. The 2nd antibody: Alexa Fluor 488-conjugated donkey anti-rabbit (1:200).

Images were taken using a Zeiss confocal laser scanning microscope (LSM700). The laser power (2%, with no fluorescence saturation under each condition) and pinhole size (1 μm) were maintained the same across different sections from individual animals. To quantify the relative pS6 levels in retrogradely labeled corticospinal neurons, we chose five sections that spanned the RFA and CFA regions (1.5 mm to −0.5 mm to the bregma, 500 μm/section) for individual animals. All images were saved as TIFF files and then blindly analyzed using ImageJ2 (NIH). On each section, we quantified the pS6 intensity in all retrogradely labeled tdTomato^+^/pS6 corticospinal neurons (∼25–40 per section). The fluorescence intensity of pS6 in individual corticospinal neurons was determined by subtracting the mean background fluorescence from the mean fluorescence intensity of pS6 fluorescence, with results expressed in arbitrary units (a.u.). Individual pS6 intensities collected from 5 sections were then averaged for each animal and used as one data point. Next, pS6 intensities in animals with different treatments were normalized to that in animals with no treatment, which was set at 1. Notably, due to the insufficiency of antibody penetration in thick brain sections, the relative expression of pS6 levels might be underestimated.

### Anterograde tracing and quantification of the CST axons

To anterogradely label the CST axons, mice received anesthesia (2% isoflurane) and were head fixed in a stereotaxic frame. We then injected an AAV that carries channelrhodopsin conjugated YFP (AAV2-ChR2-YFP, titer adjusted to 1 × 10^13^ copies/mL, generated by BrainVTA, Wuhan, China) into the RFA/CFA regions (coordinates were the same as used for intersectional labeling of corticospinal neurons). Mice received cardiac perfusion at 3 weeks post AAV-ChR2-YFP injection. Floating transverse cervical spinal sections (30 μm) were immunostained using a primary chicken anti-GFP antibody (see above) and imaged by a confocal laser-scanning microscope (Zeiss 700).

To examine the termination pattern of CST axons in the contralateral cervical spinal cord segments, we measured the relative fluorescent intensities in the dorsal (laminae II-IV), intermediate (laminae V-VI), and ventral (laminae VII-IX) spinal cord. Six to eight sections crossing the C5–C8 cervical spinal cord were used for individual animals. The laser power (2%, with no fluorescence saturation under each condition) and pinhole size (1 μm) were maintained the same across different sections from individual animals. The fluorescence intensity of YFP^+^ CST axons in different spinal laminae was determined by subtracting the mean background fluorescence from the mean fluorescence intensity of YFP fluorescence, with results expressed in arbitrary units (a.u.). Relative YFP intensities from multiple sections were first averaged for individual animals and then normalized to those of OSKTFs^Saline^ mice.

To estimate the collateral sprouting of the CST axons into the denervated side, we drew three vertical lines that evenly separated the denervated cervical spinal cord along the medial–lateral axis.^29^ For a single transverse section, we blindly quantified the number of axon fibers crossing individual vertical lines. Six to eight sections crossing the C5–C8 cervical spinal cord were used for individual animals. Next, to estimate total CST axons that were labeled at the pyramidal decussation, we imaged the medulla oblongata using a 63 × lens (Zeiss) for ChR2-YFP axons. We then randomly selected four square regions (10000 μ m^2^) within the oblongata on two consecutive sections, quantified axons within these squares, and measured the mean density of labeled axons. The total number of labeled axons = mean density × the total area. The mean values of axon fibers were then normalized to total CST axons that were quantified at the pyramidal decussation for individual animals. This method normalized anterograde labeling variability and generated the CST axon sprouting index. Indexes were then further averaged between animals with different treatments.

### Selective inactivation of corticospinal neurons with sprouted axons to the denervated spinal cord

Chemogenetic inactivation was performed in OSKTFs^Dox^ or OSKTFs^saline^ mice at 14 weeks after stroke according to previously established protocols.^25^^,^^30^ In brief, we re-performed a laminectomy and injected AAVRetro-Cre (1 × 10^13^ copies/mL generated by BrainVTA, Wuhan, China) into the denervated side of the cervical (C5–C7) spinal cord, followed by an intracortical injection of AAV-Flex-hM4Di-mCitrine (1 × 10^13^ copies/mL generated by BrainVTA, Wuhan, China) into the ipsi-lesional sensorimotor cortex. This intersectional method allows specific targeting of corticospinal neurons with midline-crossing sprouts. Three weeks later, we measured behavioral recovery before and after CNO (Sigma, C0832, 1 mg/kg in 1 × PBS, intraperitoneal) administration in mice with different treatments.

### Statistical analysis

We performed blind quantifications of all behavioral tests and histological results. For the majority of figure panels, original data points were plotted. For those without original data points, animal numbers used for statistical analysis were included in figure legends. All figure panels showing statistics were presented as mean ± standard error of the mean. We used Prism 8.0 to perform Student's *t*-test (2-tailed unpaired) for two group comparisons, and one-way ANOVA and repeated measures two-way ANOVA, both followed by Bonferroni's correction, for multiple group comparisons.

## Results

### Effects of OSKTFs expression on adult corticospinal neurons

To identify effective ways that can potentially rejuvenate corticospinal neurons, we overexpressed three transcriptional factors based on previous studies.^17^^,^^18^ To achieve this, we combined retrograde tracing with the tetracycline inducible (Tet-On) system.^31^ As the first step, we injected an AAV2Retro virus that carries a reverse tetracycline-controlled trans-activator (AAV2Retro-rtTAV16-p2A-tdTomato) into the cervical spinal cord and retrogradely labeled corticospinal neurons (Fig. 1A–C). We next injected an AAV vector that carries polycistronic Oct4-Sox2-Klf4 (referred to as OSKTFs) under the control of a tight tetracycline response element (TRE) promoter (Fig. 1A).

After an 8-week administration of doxycycline, a tetracycline derivative or saline, we performed *in situ* hybridization to examine the induction of OSKTFs in retrogradely labeled corticospinal neurons. In saline-treated animals, we observed no expression of either transcriptional factor in *tdTomato* ^*+*^ neurons (Fig. 1D–G, L, M). In contrast, doxycycline treatment induced robust expression of all three transcriptional factors in retrogradely labeled corticospinal neurons (Fig. 1H–M). OSKTFs induction did not change the identity of corticospinal neurons, since the cellular *tdTomato* signals colocalized well with those of *Fezf2* (Fig. 1G, K, N), a classic marker for identifying corticospinal neurons.^32^ Neither did it alter the density of retrogradely labeled corticospinal neurons in the sensorimotor cortex (Fig. 1O).

To examine the effects of ectopic OSKTFs expression on adult corticospinal neurons, we dissected the layer V cortical region that contained retrogradely labeled tdTomato ^+^ neurons and assessed expression of RNAs that are associated with age^33^^,^^34^ (Fig. 1P and Q). We confirmed a marked decrease in RNA levels of core histone proteins (H2A, H2B, H3, and H4) and epigenetic modulators (Lmnb1, Chaf1a, and Chaf1b) in adult mice (28 weeks) compared with P7 animals (Fig. 1R). While OSK expression did not fully restore these levels to those of P7, it significantly increased their expression relative to untreated adult controls (Fig. 1R). These findings demonstrated that OSKTFs partially reversed epigenetic aging signatures in adult corticospinal neurons.

### Corticospinal neuronal rejuvenation did not alter the CST spinal axon arborization and skilled motor function

After validating the expression of OSKTFs and their partial restoration of developmental decline in epigenetic signatures of corticospinal neurons, we first assessed whether this treatment affected the spinal projection of corticospinal neurons and the CST-dependent skilled motor function. In 20-week-old wild-type mice, we induced OSTFs expression in corticospinal neurons (Fig. 2A–C). After 8-week administration of doxycycline, we detected no deficits in either gross or skilled motor functions (Fig. 2D–F). In control mice, the CST axons were predominantly terminated in the deep dorsal horn (laminae II-IV) and intermediate zones (laminae V-VI) in the contralateral side, with few entering the ipsilateral side of the cervical spinal cord (Fig. 2G, I, J). Such termination patterns were unaltered in mice with OSKTFs treatment (Fig. 2H–J). Thus, long-term ectopic expression of OSKTFs did not change the function of mature corticospinal neurons and their termination patterns in the cervical spinal cord.

### Unilateral photothrombotic stroke ablated corticospinal neurons and compromised skilled motor function

Photothrombotic stroke enables the precise targeting of specific regions within the cerebral cortex.^20^^,^^35^ In this study, we restricted the lesion areas within the secondary and primary motor cortices that span the rostral and caudal forelimb areas (RFA & CFA), where corticospinal neurons innervating the cervical spinal cord are located^19^^,^^36^ (Fig. 3A and B).

AAV2Retro-tdTomato mediated bilateral retrograde tracing revealed that corticospinal neurons were successfully ablated within the ipsilateral RFA and CFA after photothrombotic stroke (Fig. 3C–E). In contrast, contralateral corticospinal neurons were unaffected (Fig. 3C–E). Mice with photothrombotic stroke were then subjected to behavioral measurement. In line with previous findings,^20^^,^^25^^,^^37^^,^^38^ while gross locomotion of both forepaws was minimally affected, the performance of the contra-lesional forepaw on the single pellet retrieval task and irregular ladder walking exhibited persistent impairments even at 8 weeks post stroke (Fig. 3F–H). These results highlighted that skilled motor function is dependent on the integrity of corticospinal neurons.

### Corticospinal neuron rejuvenation had moderate effects on promoting the CST axonal sprouting and skilled motor recovery post photothrombotic stroke

It has long been known that the axon sprouting capacity of the CNS declines during ageing.^39^ Therefore, we hypothesized that rejuvenation of corticospinal neurons led to enhanced collateral sprouting of the CST axons into the denervated spinal cord and thus promoted skilled locomotion following a photothrombotic stroke. To test this, we ectopically expressed OSKTFs in contra-lesional corticospinal neurons immediately post the stroke (Fig. 4A).

In line with previous findings,^20^^,^^38^ a few spontaneous midline-crossing collateral sprouting of the CST axons was observed (Fig. 4B, E). In contrast, OSKTFs treatment promoted sprouting on both intact and denervated sides (Fig. 4C–E). Behaviorally, we observed improvement of skilled motor function in animals with OSKTFs treatment at 8 weeks, but not at earlier time points post-treatment (Fig. 4F and G). Thus, rejuvenation of corticospinal neurons alone led to moderate functional recovery and circuit reorganization after a photothrombotic stroke.

### Corticospinal neuron rejuvenation enhanced the efficacy of rehabilitative training post photothrombotic stroke

Rehabilitative training is well recognized for promoting functional recovery in both human patients and animal stroke models.^40^^,^^41^ The principle underlying rehabilitative training is to promote neural plasticity, which is essential for circuit reformation.^7^^,^^40^ Given that OSKTFs treatment partially restores the developmental decline of epigenetic signatures in corticospinal neurons and may thereby counteract the associated loss of neuroplasticity, we sought to test whether this treatment could enhance the efficacy of rehabilitative training (Fig. 5A).

Consistent with previous reports,^20^^,^^38^^,^^42^ rehabilitative training of single pellet retrieval, a skilled motor function dependent on corticospinal neurons,^19^^,^^20^^,^^28^ achieved a significantly higher success rate at 8 weeks post training (Fig. 5B). Intrudingly, OSKTFs treatment further enhanced the modest effects achieved by the rehabilitative training: the success rate was higher than the control group even at 4 weeks post training and remained significantly higher at the following time points being tested (Fig. 5B). In addition, OSKTFs treatment had beneficial effects of a skilled locomotion task among untrained mice at 8 weeks post treatment (Fig. 5C), probably due to enhanced neuroplasticity.

We went further to examine whether OSKTFs treatment had any effects on the collateral sprouting of the CST axons in the spinal cord. As shown in Figure 5D, E, G, rehabilitative training led to significant sprouting of the intact CST axons into the denervated spinal cord. OSKTFs treatment further amplified the promoting effects achieved by rehabilitative training, resulting in more robust CST axon sprouting (Fig. 5D, F, G). Taken together, these results demonstrated that ectopic expression of OSKTFs in corticospinal neurons significantly enhanced neuroplasticity and thus the efficacy of task-dependent rehabilitative training in adult animals.

### Rejuvenation of corticospinal neurons amplified their mTOR activity evoked by rehabilitative training post photothrombotic stroke

To investigate the cellular mechanisms underlying the combinatory treatment of OSKTFs and rehabilitative training, we examined the mTOR activity in corticospinal neurons, which is known to be associated with enhanced cortical activity and CST axon regrowth after traumatic CNS injuries.^29^^,^^43^^,^^44^

Normalized immunofluorescence of phosphorylated ribosomal protein S6 (pS6), a well-defined indicator of mTOR activation,^29^^,^^45^ exhibited a moderate increment in mice receiving rehabilitative training (Fig. 6A, B, E). Intriguingly, ectopic expression of OSKTFs evoked even greater expression of pS6 in corticospinal neurons (Fig. 6C, E), a feature seen in young layer V cortical neurons.^29^ The OSKTFs overexpression-induced pS6 up-regulation was further amplified when it was paired with rehabilitative training (Fig. 6D and E), indicative of synergistic effects in activating corticospinal neurons for axon sprouting.

### mTOR- and sprouted CST axon-dependent recovery following combined treatment after photothrombotic stroke

To determine whether mTOR activation is required for CST axon sprouting and behavioral recovery, we administered rapamycin, a classic mTOR inhibitor,^46^ during OSKTF induction (Fig. 7A). Rapamycin treatment largely abolished the promoting effects of combined treatment on CST collateral sprouting and functional recovery (Fig. 7B–G), demonstrating that mTOR activation is required for these outcomes. Thus, even in rejuvenated corticospinal neurons, mTOR signaling remains essential for axon sprouting and functional recovery.

We next tested whether behavioral improvement was mediated by CST axonal sprouting. To this end, we injected AAVRetro-Cre into the denervated cervical spinal cord and AAV-Flex-GFP or hM4Di-mCitrine into the intact sensorimotor cortex (Fig. 7H and I). This intersectional approach enables selective expression of inhibitory chemogenetic receptors (hM4Di)^47^ in corticospinal neurons with midline-crossing sprouts (Fig. 7J). In control animals expressing GFP, CNO administration had minimal effects on skilled forelimb recovery (Fig. 7K and L). In sharp contrast, in animals with hM4Di-expressing sprouted CST neurons, CNO treatment significantly impaired recovered forelimb function (Fig. 7K and L). Thus, the behavioral recovery induced by OSKTFs is critically dependent on CST axonal sprouting driven by mTOR activation.

## Discussion

Both the axon sprouting capacity and the efficacy of rehabilitative training following traumatic CNS injuries significantly decline during development.^39^^,^^48^ To overcome age-associated reduction of neuroplasticity, we made efforts to identify strategies for rejuvenating adult corticospinal neurons. Our results showed that ectopic expression of OSKTFs reset epigenetic ageing signatures in adult corticospinal neurons without affecting their neuronal identity and function in modulating skilled locomotion. OSKTFs treatment alone had moderate effects on promoting collateral sprouting of the CST axons and skilled motor function after stroke. In contrast, combining OSKTFs with rehabilitative training produced synergistic effects, markedly enhancing CST axonal sprouting and behavioral recovery. Mechanistically, these benefits required mTOR activation and were mediated by midline-crossing corticospinal sprouts. Our results thus provided novel strategies to rejuvenate adult corticospinal neurons and, more importantly, avenues to promote functional recovery post-traumatic CNS injuries.

Recent studies revealed that systematic delivery of AAV-mediated OSKTFs partially altered patterns of DNA methylation, an epigenetic mark, shifting them from an aged to a more youthful state across various cell types.^17^^,^^33^^,^^49^ Our study showed that ectopic expression of OSKTFs effectively rescued the drastic postnatal loss of histone proteins and epigenetic modulators in adult corticospinal neurons. Phenotypically, OSKTFs treatment enhanced neuroplasticity and restored intracellular mTOR activity, which normally declines during development.^29^ While our findings demonstrate that OSKTFs reverse developmental declines in regenerative capacity, it remains unknown whether, and to what extent, the same treatment could reverse changes associated with chronological aging in corticospinal neurons. Intriguingly, ectopic expression of OSKTFs did not change the spinal projection patterns of corticospinal neurons, nor did it impact skilled locomotion, suggesting that the neuronal identity of corticospinal neurons was unaffected. In contrast, ectopic expression of Febf2 in layer II/III callosal projection neurons leads to direct lineage reprogramming, converting those neurons into layer V/IV corticofugal neurons.^50^

Accumulating evidence showed that younger individuals maintained greater neuroplasticity and healing potential.^6^^,^^9^ For instance, neonatal rats that underwent unilateral motor cortex removal displayed substantial sprouting of intact corticofugal axons, along with proficient skilled motor function 10 weeks later, a level of recovery that has not been seen in adult rats with similar injuries.^8^^,^^51^ In contrast, aged rats subjected to a lesion in the forelimb area showed no improvement in sensorimotor function over 4 weeks post-injury.^52^ Although OSKTFs treatment increased the collateral sprouting potential, as shown by enhanced pS6 levels, the effects remain only partial. One possible explanation is that individual factors within the OSK cocktail may exert opposing influences on the axonal growth of corticospinal neurons. For example, KLF4 has been reported to suppress neurite extension in cultured cortical neurons,^53^ suggesting that alternative combinations of factors could be more effective for promoting CST plasticity than OSKTFs. In addition, global neural activity patterns in the brain and spinal cord differ markedly between neonatal and adult animals, which may further limit the efficacy of OSKTFs alone. Together, these considerations highlight the need for future studies that systematically compare factor combinations with both poly- or monocistronic overexpression and integrate OSKTFs expression with complementary strategies to optimize CST sprouting.

Rehabilitative training fosters the probability of simultaneous pre- and postsynaptic activities, thereby providing better chances for newly sprouted axon terminals to form synapses with spinal interneurons. However, rehabilitative training evoked plasticity also drastically declines during aging. For instance, Tennant et al showed that while task-dependent rehabilitative training leads to functional recovery, the reorganization of neural circuits is highly constrained by age.^48^ Therefore, rejuvenating corticospinal neurons might benefit both spontaneous and rehabilitative training-induced neuroplasticity, resulting in synergetic effects on promoting axonal outgrowth and functional recovery.

Our findings indicate that mTOR activation is a key mediator of OSKTFs-induced CST sprouting and functional recovery. Pharmacological inhibition with rapamycin abolished both axonal outgrowth and behavioral improvements, supporting an essential role for mTOR signaling in this process. Nonetheless, pharmacological evidence alone is not sufficient to establish definitive causality. Future studies employing genetic loss-of-function strategies, such as disruption of mTOR, Rptor, or downstream effectors in corticospinal neurons, would provide more conclusive evidence for this mechanism. Moreover, a more comprehensive understanding of how OSKTFs remodel the transcriptional and epigenetic landscape to promote sprouting will require single-cell RNA sequencing and ATAC sequencing coupled with functional validation.

AAV-mediated intersectional expression of OSKTFs in corticospinal neurons is less translatable. However, a recent study showed that a six-chemical cocktail mimicked the effects of OSKTFs in reversing transcriptomic age without compromising cell identities.^54^ Thus, it will be promising to combine new avenues that are more translational with rehabilitative training to achieve optimal effects on improving functional recovery post-traumatic brain and spinal cord injuries.

## CRediT authorship contribution statement

**Bin Sun:** Methodology, Investigation, Formal analysis, Data curation, Conceptualization. **Li Sun:** Software, Methodology, Investigation, Formal analysis, Data curation. **Lixiang Zhang:** Formal analysis, Data curation. **Xinyu Xue:** Visualization, Validation, Software, Resources. **Qiuyan Tian:** Software, Resources, Formal analysis, Data curation. **Lei Wu:** Formal analysis, Data curation. **Mei Li:** Software, Resources, Methodology. **Jian Huang:** Resources, Formal analysis. **Hong Ni:** Methodology, Investigation, Formal analysis. **Lixiao Xu:** Project administration, Methodology, Investigation, Funding acquisition, Formal analysis, Data curation. **Chenxi Feng:** Visualization, Validation, Supervision, Software. **Jing Ren:** Software, Resources, Project administration. **Hongliang Huo:** Project administration, Methodology, Investigation, Conceptualization. **Xia Zhang:** Visualization, Validation, Supervision. **Xing Feng:** Writing – original draft, Visualization, Validation, Supervision, Funding acquisition. **Wenhao Zhou:** Validation, Supervision, Resources, Project administration. **Wanliang Guo:** Investigation, Formal analysis, Data curation. **Yaobo Liu:** Supervision, Software. **Rong Ju:** Resources. **Zhenlang Lin:** Supervision, Software, Data curation. **Xiaofeng Yang:** Writing – review & editing, Writing – original draft, Visualization, Funding acquisition. **Xin Ding:** Writing – review & editing, Writing – original draft, Supervision, Investigation, Funding acquisition, Conceptualization.

## Ethics declaration

All animal surgeries and procedures were approved by the ethical committee of Soochow University (No. XD-2020-1) and performed in accordance with the institution's guidelines for animal use and care.

## Data availability

All original data will be made available on reasonable request to Xin Ding.

## Funding

This work is funded by the 10.13039/501100001809National Natural Science Foundation of China (No. 82471742 to X.D., 82271739 to X.D., 82171703 to X.F., 82271405 to M.L.); the National Key R&D Program Project (China) (No. 2024YFC2707700 to W.H.Z.); the Natural Science Foundation of Jiangsu Province, China (No. BK20200207 to L.X.X.); the Jiangsu Provincial Key Medical Discipline (China) (No. ZDXKA2016013 to X.F.); the Training Program Foundation for Health Talents of Gusu (China) (No. GSWS2020052 to X.D., GSWS2019049 to X.D.); the Project of Suzhou Science and Technology Development Plan (China) (No. SKY2021008 to B.S., SYS2020154 to C.X.F.); and the Suzhou Science, Education and Health and Technology Project (China) (No. KJXW2018018 to X.D.).

## Conflict of interests

The authors declared no competing interests.
