# Supplementary material for: Rejuvenation of corticospinal neurons enhances rehabilitation-associated corticospinal tract axon sprouting and functional recovery post photothrombotic ischemic stroke in mice
Source: Genes Dis. 2025 Dec 22;13(5):102000. doi: 10.1016/j.gendis.2025.102000 (PMC13285357; doi:10.1016/j.gendis.2025.102000)
Supplement: Multimedia component 1 [file mmc1.docx]

**Supplementary Fig. 1:** **AAV vector** **sequence and map for the TRE-OSK construct**

**Map:**


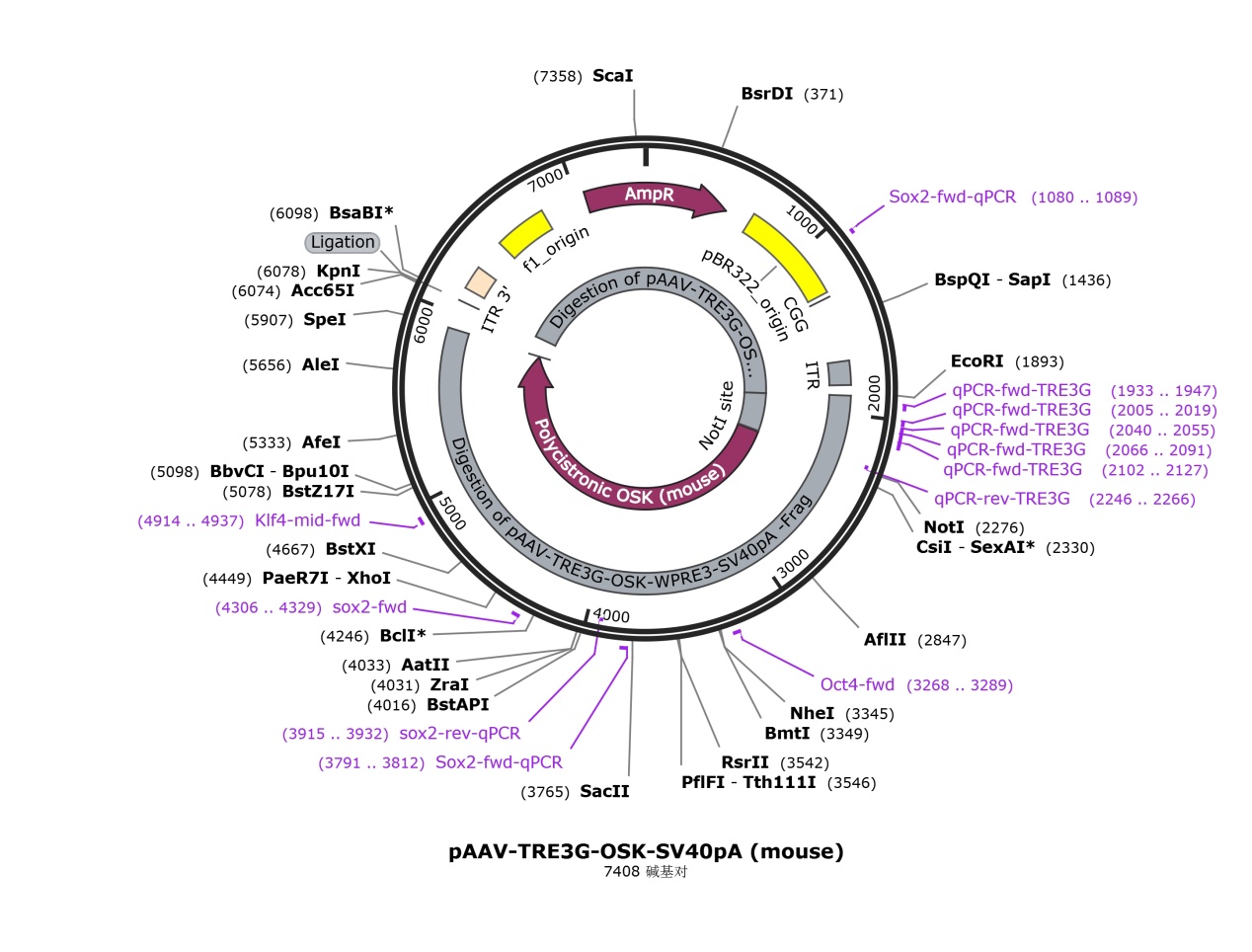


**Sequence:**

ttatgcagtgctgccataaccatgagtgataacactgcggccaacttacttctgacaacgatcggaggaccgaaggagctaaccgcttttttgcacaacatgggggatcatgtaactcgccttgatcgttgggaaccggagctgaatgaagccataccaaacgacgagcgtgacaccacgatgcctgtagtaatggtaacaacgttgcgcaaactattaactggcgaactacttactctagcttcccggcaacaattaatagactggatggaggcggataaagttgcaggaccacttctgcgctcggcccttccggctggctggtttattgctgataaatctggagccggtgagcgtgggtctcgcggtatcattgcagcactggggccagatggtaagccctcccgtatcgtagttatctacacgacggggagtcaggcaactatggatgaacgaaatagacagatcgctgagataggtgcctcactgattaagcattggtaactgtcagaccaagtttactcatatatactttagattgatttaaaacttcatttttaatttaaaaggatctaggtgaagatcctttttgataatctcatgaccaaaatcccttaacgtgagttttcgttccactgagcgtcagaccccgtagaaaagatcaaaggatcttcttgagatcctttttttctgcgcgtaatctgctgcttgcaaacaaaaaaaccaccgctaccagcggtggtttgtttgccggatcaagagctaccaactctttttccgaaggtaactggcttcagcagagcgcagataccaaatactgtccttctagtgtagccgtagttaggccaccacttcaagaactctgtagcaccgcctacatacctcgctctgctaatcctgttaccagtggctgctgccagtggcgataagtcgtgtcttaccgggttggactcaagacgatagttaccggataaggcgcagcggtcgggctgaacggggggttcgtgcacacagcccagcttggagcgaacgacctacaccgaactgagatacctacagcgtgagctatgagaaagcgccacgcttcccgaagggagaaaggcggacaggtatccggtaagcggcagggtcggaacaggagagcgcacgagggagcttccagggggaaacgcctggtatctttatagtcctgtcgggtttcgccacctctgacttgagcgtcgatttttgtgatgctcgtcaggggggcggagcctatggaaaaacgccagcaacgcggcctttttacggttcctggccttttgctggccttttgctcacatgttctttcctgcgttatcccctgattctgtggataaccgtattaccgcctttgagtgagctgataccgctcgccgcagccgaacgaccgagcgcagcgagtcagtgagcgaggaagcggaagagcgcccaatacgcaaaccgcctctccccgcgcgttggccgattcattaatgcagctggcacgacaggtttcccgactggaaagcgggcagtgagcgcaacgcaattaatgtgagttagctcactcattaggcaccccaggctttacactttatgcttccggctcgtatgttgtgtggaattgtgagcggataacaatttcacacaggaaacagctatgaccatgattacgccagatttaattaaggccttaattaggctgcgcgctcgctcgctcactgaggccgcccgggcaaagcccgggcgtcgggcgacctttggtcgcccggcctcagtgagcgagcgagcgcgcagagagggagtggccaactccatcactaggggttccttgtagttaatgattaacccgccatgctacttatctacgtagccatgctctaggaagatcggaattctttactccctatcagtgatagagaacgtatgaagagtttactccctatcagtgatagagaacgtatgcagactttactccctatcagtgatagagaacgtataaggagtttactccctatcagtgatagagaacgtatgaccagtttactccctatcagtgatagagaacgtatctacagtttactccctatcagtgatagagaacgtatatccagtttactccctatcagtgatagagaacgtataagctttaggcgtgtacggtgggcgcctataaaagcagagctcgtttagtgaaccgtcagatcgcctggagcaattccacaacacttttgtcttataccaactttccgtaccacttcctaccctcgtaaagcggccgcgccaccatggctggacacctggcttcagacttcgccttctcacccccaccaggtgggggtgatgggtcagcagggctggagccgggctgggtggatcctcgaacctggctaagcttccaagggcctccaggtgggcctggaatcggaccaggctcagaggtattggggatctccccatgtccgcccgcatacgagttctgcggagggatggcatactgtggacctcaggttggactgggcctagtcccccaagttggcgtggagactttgcagcctgagggccaggcaggagcacgagtggaaagcaactcagagggaacctcctctgagccctgtgccgaccgccccaatgccgtgaagttggagaaggtggaaccaactcccgaggagtcccaggacatgaaagccctgcagaaggagctagaacagtttgccaagctgctgaagcagaagaggatcaccttggggtacacccaggccgacgtggggctcaccctgggcgttctctttggaaaggtgttcagccagaccaccatctgtcgcttcgaggccttgcagctcagccttaagaacatgtgtaagctgcggcccctgctggagaagtgggtggaggaagccgacaacaatgagaaccttcaggagatatgcaaatcggagaccctggtgcaggcccggaagagaaagcgaactagcattgagaaccgtgtgaggtggagtctggagaccatgtttctgaagtgcccgaagccctccctacagcagatcactcacatcgccaatcagcttgggctagagaaggatgtggttcgagtatggttctgtaaccggcgccagaagggcaaaagatcaagtattgagtattcccaacgagaagagtatgaggctacagggacacctttcccagggggggctgtatcctttcctctgcccccaggtccccactttggcaccccaggctatggaagcccccacttcaccacactctactcagtcccttttcctgagggcgaggcctttccctctgttcccgtcactgctctgggctctcccatgcattcaaacgctagcggcagcggcgccacgaacttctctctgttaaagcaagcaggagatgttgaagaaaaccccgggcctgcatgcatgtataacatgatggagacggagctgaagccgccgggcccgcagcaagcttcggggggcggcggcggaggaggcaacgccacggcggcggcgaccggcggcaaccagaagaacagcccggaccgcgtcaagaggcccatgaacgccttcatggtatggtcccgggggcagcggcgtaagatggcccaggagaaccccaagatgcacaactcggagatcagcaagcgcctgggcgcggagtggaaacttttgtccgagaccgagaagcggccgttcatcgacgaggccaagcggctgcgcgctctgcacatgaaggagcacccggattataaataccggccgcggcggaaaaccaagacgctcatgaagaaggataagtacacgcttcccggaggcttgctggcccccggcgggaacagcatggcgagcggggttggggtgggcgccggcctgggtgcgggcgtgaaccagcgcatggacagctacgcgcacatgaacggctggagcaacggcagctacagcatgatgcaggagcagctgggctacccgcagcacccgggcctcaacgctcacggcgcggcacagatgcaaccgatgcaccgctacgacgtcagcgccctgcagtacaactccatgaccagctcgcagacctacatgaacggctcgcccacctacagcatgtcctactcgcagcagggcacccccggtatggcgctgggctccatgggctctgtggtcaagtccgaggccagctccagcccccccgtggttacctcttcctcccactccagggcgccctgccaggccggggacctccgggacatgatcagcatgtacctccccggcgccgaggtgccggagcccgctgcgcccagtagactgcacatggcccagcactaccagagcggcccggtgcccggcacggccattaacggcacactgcccctgtcgcacatggcatgcggctccggcgagggcaggggaagtcttctaacatgcggggacgtggaggaaaatcccggcccactcgagatgaggcagccacctggcgagtctgacatggctgtcagcgacgctctgctcccgtccttctccacgttcgcgtccggcccggcgggaagggagaagacactgcgtccagcaggtgccccgactaaccgttggcgtgaggaactctctcacatgaagcgacttcccccacttcccggccgcccctacgacctggcggcgacggtggccacagacctggagagtggcggagctggtgcagcttgcagcagtaacaacccggccctcctagcccggagggagaccgaggagttcaacgacctcctggacctagactttatcctttccaactcgctaacccaccaggaatcggtggccgccaccgtgaccacctcggcgtcagcttcatcctcgtcttccccagcgagcagcggccctgccagcgcgccctccacctgcagcttcagctatccgatccgggccgggggtgacccgggcgtggctgccagcaacacaggtggagggctcctctacagccgagaatctgcgccacctcccacggcccccttcaacctggcggacatcaatgacgtgagcccctcgggcggcttcgtggctgagctcctgcggccggagttggacccagtatacattccgccacagcagcctcagccgccaggtggcgggctgatgggcaagtttgtgctgaaggcgtctctgaccacccctggcagcgagtacagcagcccttcggtcatcagtgttagcaaaggaagcccagacggcagccaccccgtggtagtggcgccctacagcggtggcccgccgcgcatgtgccccaagattaagcaagaggcggtcccgtcctgcacggtcagccggtccctagaggcccatttgagcgctggaccccagctcagcaacggccaccggcccaacacacacgacttccccctggggcggcagctccccaccaggactacccctacactgagtcccgaggaactgctgaacagcagggactgtcaccctggcctgcctcttcccccaggattccatccccatccggggcccaactaccctcctttcctgccagaccagatgcagtcacaagtcccctctctccattatcaagagctcatgccaccgggttcctgcctgccagaggagcccaagccaaagaggggaagaaggtcgtggccccggaaaagaacagccacccacacttgtgactatgcaggctgtggcaaaacctataccaagagttctcatctcaaggcacacctgcgaactcacacaggcgagaaaccttaccactgtgactgggacggctgtgggtggaaattcgcccgctccgatgaactgaccaggcactaccgcaaacacacagggcaccggccctttcagtgccagaagtgcgacagggccttttccaggtcggaccaccttgccttacacatgaagaggcactaaatgactagtgcgcgcagcggccgaccatggcccaacttgtttattgcagcttataatggttacaaataaagcaatagcatcacaaatttcacaaataaagcatttttttcactgcattctagttgtggtttgtccaaactcatcaatgtatcttatcatgtctggatctcggtaccggatccaaattcccgataaggatcttcctagagcatggctacgtagataagtagcatggcgggttaatcattaactacaaggaacccctagtgatggagttggccactccctctctgcgcgctcgctcgctcactgaggccgggcgaccaaaggtcgcccgacgcccgggctttgcccgggcggcctcagtgagcgagcgagcgcgcagccttaattaacctaattcactggccgtcgttttacaacgtcgtgactgggaaaaccctggcgttacccaacttaatcgccttgcagcacatccccctttcgccagctggcgtaatagcgaagaggcccgcaccgatcgcccttcccaacagttgcgcagcctgaatggcgaatgggacgcgccctgtagcggcgcattaagcgcggcgggtgtggtggttacgcgcagcgtgaccgctacacttgccagcgccctagcgcccgctcctttcgctttcttcccttcctttctcgccacgttcgccggctttccccgtcaagctctaaatcgggggctccctttagggttccgatttagtgctttacggcacctcgaccccaaaaaacttgattagggtgatggttcacgtagtgggccatcgccctgatagacggtttttcgccctttgacgttggagtccacgttctttaatagtggactcttgttccaaactggaacaacactcaaccctatctcggtctattcttttgatttataagggattttgccgatttcggcctattggttaaaaaatgagctgatttaacaaaaatttaacgcgaattttaacaaaatattaacgtttataatttcaggtggcatctttcggggaaatgtgcgcggaacccctatttgtttatttttctaaatacattcaaatatgtatccgctcatgagacaataaccctgataaatgcttcaataatattgaaaaaggaagagtatgagtattcaacatttccgtgtcgcccttattcccttttttgcggcattttgccttcctgtttttgctcacccagaaacgctggtgaaagtaaaagatgctgaagatcagttgggtgcacgagtgggttacatcgaactggatctcaatagtggtaagatccttgagagttttcgccccgaagaacgttttccaatgatgagcacttttaaagttctgctatgtggcgcggtattatcccgtattgacgccgggcaagagcaactcggtcgccgcatacactattctcagaatgacttggttgagtactcaccagtcacagaaaagcatcttacggatggcatgacagtaagagaa
